# Strengthened Corticosubcortical Functional Connectivity during Muscle Fatigue

**DOI:** 10.1155/2016/1726848

**Published:** 2016-10-17

**Authors:** Zhiguo Jiang, Xiao-Feng Wang, Guang H. Yue

**Affiliations:** ^1^Human Performance and Engineering Research, Kessler Foundation, 1199 Pleasant Valley Way, West Orange, NJ, USA; ^2^Department of Quantitative Health Science, The Cleveland Clinic, 9500 Euclid Avenue/JJN3, Cleveland, OH, USA; ^3^Department of Biomedical Engineering, New Jersey Institute of Technology, 323 Martin Luther King, Jr. Boulevard, Newark, NJ, USA; ^4^Department of Physical Medicine & Rehabilitation, Rutgers New Jersey Medical School, 185 S. Orange Ave, Newark, NJ, USA

## Abstract

The present study examined functional connectivity (FC) between functional MRI (fMRI) signals of the primary motor cortex (M1) and each of the three subcortical neural structures, cerebellum (CB), basal ganglia (BG), and thalamus (TL), during muscle fatigue using the quantile regression technique. Understanding activation relation between the subcortical structures and the M1 during prolonged motor performance should help delineate how central motor control network modulates acute perturbations at peripheral sensorimotor system such as muscle fatigue. Ten healthy subjects participated in the study and completed a 20-minute intermittent handgrip motor task at 50% of their maximal voluntary contraction (MVC) level. Quantile regression analyses were carried out to compare the FC between the contralateral (left) M1 and CB, BG, and TL in the minimal (beginning 100 s) versus significant (ending 100 s) fatigue stages. Widespread, statistically significant increases in FC were found in bilateral BG, CB, and TL with the left M1 during significant versus minimal fatigue stages. Our results imply that these subcortical nuclei are critical components in the motor control network and actively involved in modulating voluntary muscle fatigue, possibly, by working together with the M1 to strengthen the descending central command to prolong the motor performance.

## 1. Introduction

Muscle fatigue is associated with a decrease in the ability of the involved muscle to produce force in order to maintain the same force or resist against the same load (such as carrying a suitcase for a relatively long time). Under the fatigue condition, additional motor units/muscle fibers need to be recruited and/or their activation level increased to compensate for the loss of force in the fatigued motor units provided that the sustained force/load is submaximal (when producing maximal forces, all motor units that can voluntarily be recruited are already active). Increased activation level has been observed in many brain regions using functional magnetic resonance imaging (fMRI) during fatigue [1, 2] and the elevated brain activation accompanied accretion of electromyogram (EMG) activities of the contracting muscles (a reflection of motor unity activity level) [1]. These fMRI data indicate augmented effort by the central nervous system (CNS) to sustain the motor task via sending stronger command through the descending motor pathway for the aforementioned motor unit recruitment and/or elevation of motor unit activation level under fatigue condition. One way to strengthen the descending command is to synchronize activities of multiple cortical and subcortical areas that contribute to the corticospinal output to the spinal motor neuron pool and the synchronization can be estimated by determining the level of functional connectivity (FC, cross-correlation) among the active motor control fields. Indeed, we have demonstrated increased FC among many cortical regions including primary motor cortices (M1), primary sensory cortices (S1), premotor cortices (PMC), supplementary motor area (SMA), and prefrontal cortices (PFC) based on fMRI data, during progressive muscle fatigue [3].

While these cortical fields play important roles in motor control, voluntary movements cannot be properly accomplished without active participation by their subcortical counterparts including cerebellum (CB), basal ganglia (BG), and thalamus (TL). For example, the BG and the CB are connected to cortical control network through the TL [4] to form a motor control circuit [5], which is critical for planning and executing of any voluntary movements (for a comprehensive understanding of the role of CB, BG, and TL and their relation to the M1 and other higher-order cortices in motor control, see Kandel et al. [6]). Impairments to the BG have been linked to movement disorders such as Parkinson's and Huntington disease [7, 8]. Injury or disease in the CB is known to affect fine control of movement [9]. Defection in the TL has been connected to dystonia and dyskinesia [10]. Motor units recruitment by the M1 can be upregulated during fatiguing endurance exercises by brain circuitry involving the BG and the TL [11]. Activation of the CB has been shown to increase with the M1 during muscle fatigue [1]. Considering their important roles in the motor control network, we reasoned that these subcortical nuclei would act similarly to the cortical motor centers to deal with muscle fatigue-related force loss by increasing their FC with the M1, presumably for the purpose of strengthening the descending command. There has been evidence supporting regulatory roles of BG, CB, and TL under fatigue conditions [12]. However, the relationship between activities of these three subcortical structures and those exhibited by the contralateral M1 to the performing limb during a fatigue process remains unexplored. Furthermore, many clinical populations exhibit debilitating feeling of fatigue that compromises their quality of life and the primary mechanism behind the fatigue is of central origin (as patients feel fatigued even without doing physical activities) that most likely involves both the cortical and subcortical motor control circuits. Thus, a better understanding of how the subcortical network responds to muscle fatigue together with the M1 in health would help decipher contributing factors to worsened feeling of fatigue during motor performance in disease (e.g., individuals with multiple sclerosis and cancer survivors). The purpose of this study was to estimate FC between the fMRI time course signal of the M1 and that of the BG, CB, and TL during a prolonged handgrip task. We hypothesized that the FC between the subcortical regions and contralateral M1 would show fatigue-related increases. The information is potentially important for understanding the roles of cortical and subcortical contributions to integrated control of muscle activities under fatigue condition for the purpose of optimizing motor performance.

## 2. Material and Methods

### 2.1. Subjects and Motor Task

Ten healthy subjects participated in this study (age = 32.8 ± 8.4 years, all males). All subjects were right handed based on the Edinburgh inventory [13]. None of the subjects had a history of neurological or psychiatric disorder. Written informed consents were obtained from all subjects and all procedures were approved by the local Institutional Review Board.

All subjects were instructed to perform an intermittent handgrip contraction task at 50% maximal voluntary contraction (MVC) force level (determined right before the task) using the right hand while their brains were scanned continuously. The task paradigm employed a repeated block design (On and Off blocks) as described previously in more detail [3]. The submaximal contractions were performed for 3.5 s each (On blocks) followed by a 6.5 s rest (Off blocks) and the entire motor task lasted 20 minutes (consisting of 120 handgrip trials). The MVC force was measured both before and immediately after the last handgrip to determine the level of fatigue after completing the motor task.

### 2.2. MRI Acquisition

We collected all MRI scans using a 3T Siemens Trio scanner (Siemens, Germany) while subjects remained in supine position throughout the scan sessions. Subjects were instructed to remain still during the task and foam pads were used to help stabilize their head to minimize excessive motion. Functional images were acquired using a T2^*∗*^ weighted echo planar sequence. The parameters were as follows: repetition time (TR)/echo time (TE) = 2000/30 ms; flip angle = 50°; slice thickness = 4 mm; and in-plane resolution = 3.44 mm × 3.44 mm. A total of 30 slices were collected to cover the entire brain. A high-resolution T1 weighted magnetization prepared rapid gradient echo (MPRAGE) scan was also acquired on sagittal plane with the following parameters: TR = 2600 ms; TE = 3.93 ms; FOV = 256 m × 256 mm; slice thickness = 1 mm; and in-plane resolution = 1 mm × 1 mm.

### 2.3. FC Analysis

All data was processed using BrainVoyager QX 1.7 (Brain Innovation, http://www.brainvoyager.com/). Preprocessing included slice time correction and motion correction and linear trend removal and normalization and smoothing in the same manner as described previously [3]. The MPRAGE scans were transformed into the standard Talairach space and functional data were coregistered to their corresponding MPRAGE scans. The registration information was used later to project functional data into common Talairach space to enable group level analysis.

In order to investigate the effect of fatigue on FC, we first defined the minimal and significant fatigue stage as the first 100 s and the last 100 s of the motor task, respectively. The seed used for the FC analysis was determined by finding the local signal maxima within the contralateral (left) primary motor cortex (M1) which showed the most significant correlation to an ideal time course (defined by a 0 for each Off time point and a 1 for each On time point in the contraction). FC maps were created for both fatigue stages by computing the Pearson correlation coefficients between the local fMRI time courses and the seed time course for all voxels in the entire brain.

### 2.4. Region of Interest (ROI)

In order to delineate the subcortical structures from high-resolution T1 scans, we used public domain software Freesurfer V5.1 (http://surfer.nmr.mgh.harvard.edu/) to segment the structural data. Each voxel in the T1 volume was assigned one of the 45 labels (for a complete list, refer to http://freesurfer.net/fswiki/SubcorticalSegmentation). These label maps were then projected back to fMRI data using the obtained coregistration information in preprocessing to define ROIs to extract the voxel data from corresponding FC maps. Labels denoting the basal ganglia (caudate, putamen, and pallidum) and thalamus were selected as ROIs for the analysis. The cerebellum was divided bilaterally and analyzed accordingly based on the AAL atlas [14].

### 2.5. Statistical Analyses

#### 2.5.1. Quantile Regression

We used quantile regression to compare the correlation coefficients between the two (minimal and significant) fatigue stages. Quantile regression is a powerful statistic tool for analyzing relationships between variables using quantiles. Quantile regression extends the conventional regression model (which utilizes the mean value) to different quantiles of the data. This provides us with a more complete view of the relationship of the two variables in different quantiles instead of just looking at the means (e.g., Student *t*-test only examines if there is a significant difference between the means of two groups). Quantile regression analysis has previously been successfully applied to the heterogeneous fMRI data by our group [15] and shown to be able to capture more dynamic changes which can be overlooked by using conventional ANOVA models [3]. All quantile regression analyses were done using the quantile regression package in R (https://www.r-project.org/).

#### 2.5.2. Standard Statistical Analyses

MVC forces before and after the fatigue task were assessed using paired-sample *t*-test. Differences between the means of FC measured in the minimal and significant fatigue stages were compared using quantile regression and 1-way ANOVA within each defined ROI. Significance level was set at 0.05 for all the analyses (quantile regression, *t*, and ANOVA).

## 3. Results

Immediately after the fatigue task (120 handgrip contractions at 50% MVC), the handgrip MVC force declined significantly (31.9 ± 15.5%, mean ± SD, *P* < 0.01) compared with the prefatigue task MVC force. The significant decrease in the MVC force indicates muscle fatigue after performing the 120 contractions. When fatigue develops, force generating capability of the affected motor units declines and the brain has to compensate for this deficit by recruiting additional motor units and/or increase activation level of the active motor units to prolong the same motor task with a given load or resistance [16]. Indeed, this increase of central drive has been manifested by observed increases in brain activation level and voluntary EMG amplitude [1] and FC between the M1 and other cortical regions [3].

**Figure 1:**
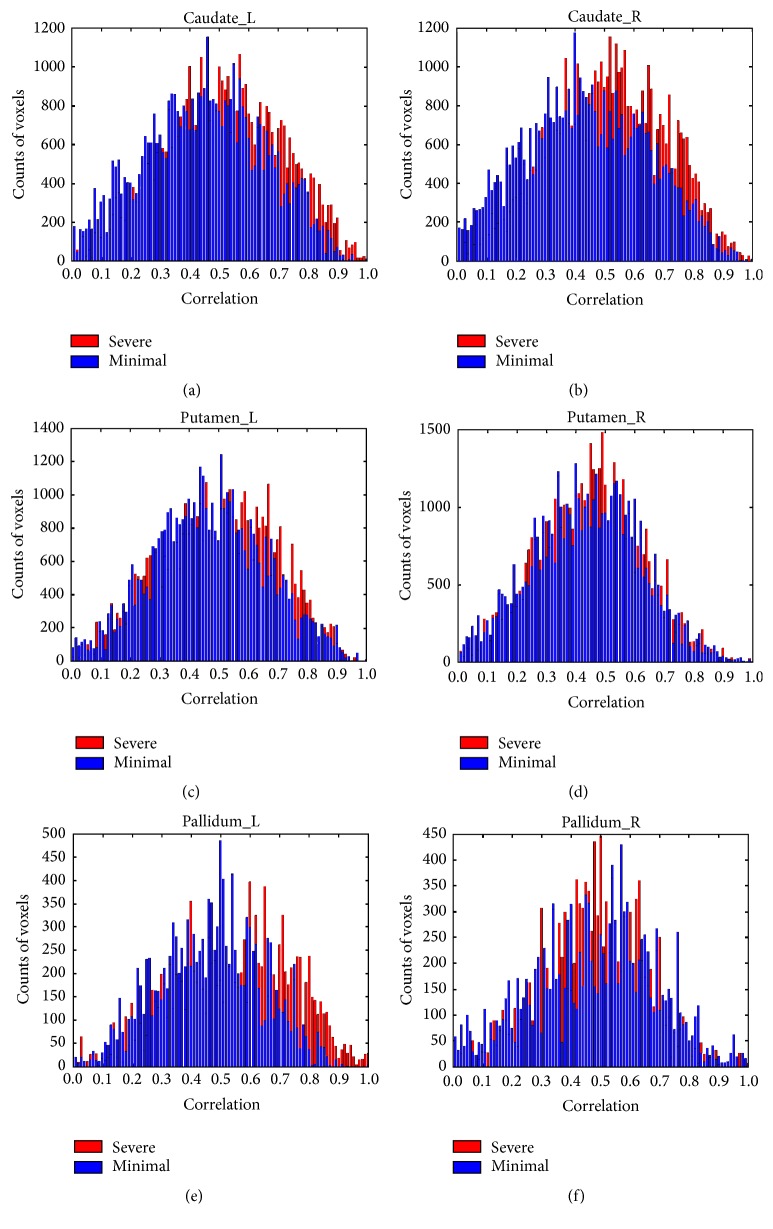
Comparisons of histogram plots of correlation coefficients over all subjects in the bilateral basal ganglia (caudate, putamen, and pallidum). Blue bins = data for* minimal* fatigue stage. Red bins = data for* significant* fatigue stage. Voxels with very low correlation (*r* < 0.001) were excluded for better visualization of coefficient distribution of the voxels in each ROI. In the significant fatigue stage, a larger number of voxels (red bins) can be seen with higher correlation (FC with seed area in M1) values in mid-to-high correlation range in all basal ganglia ROIs.

**Figure 2:**
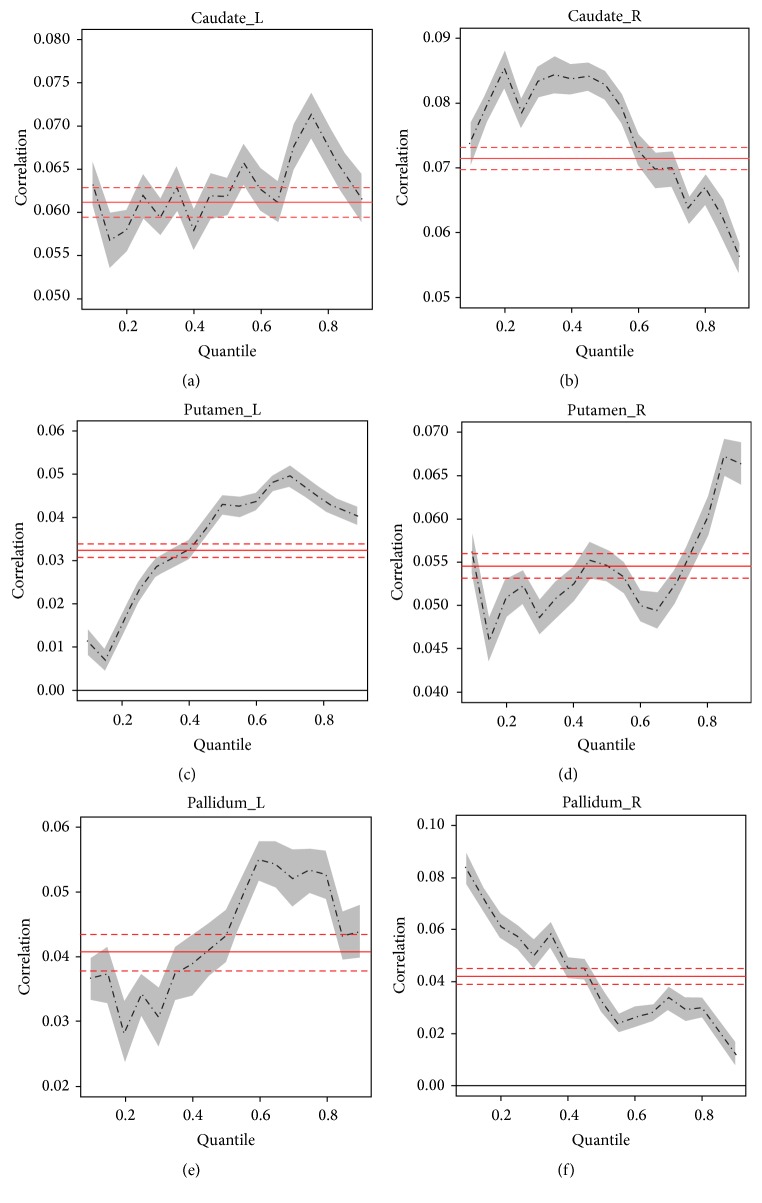
Results of quantile analysis from comparisons of coefficients of cross-correlation (FC) between minimal (first 100 s) and significant (last 100 s) fatigue stages in bilateral caudate, putamen, and pallidum. Comparisons were made at each quantile of the distribution curve between minimal and significant fatigue conditions in each ROI. ANOVA results were plotted in red (solid line = mean; dotted line = 95% confidential intervals).

We found significant increases of FC in all BG subregions in significant fatigue stage comparing to minimal fatigue stage.  Figure 1 shows the histogram (bins with *r* < 0.001 representing a huge number of voxels in nonmotor regions whose activities did not correlate with the M1 signal were excluded) plots of FC data for all bilateral BG subregions. The histogram showed a remarkable “shift” of FC to higher correlation for the majority of the ROIs (bilateral caudate, left putamen, and left pallidum) in the significant fatigue stage. More voxels (denoted by taller bins) turned out on right tail (higher *r* values) of the histograms (especially in bilateral caudate and left pallidum) during significant (last 100 s) versus minimal (first 100 s) fatigue stages (Figure 1). Quantile regression analysis confirmed that the FC increased significantly in significant fatigue stage compared to minimal fatigue stage in bilateral caudate, putamen, and pallidum with M1 as shown in Figure 2. Both standard ANOVA and quantile regression analysis were performed to evaluate the magnitude of FC during performance of the intermittent motor task. Figure 2 shows the ANOVA and quantile analysis results from comparison of FC of the minimal to that of significant fatigue stages. In each plot, the red solid line denotes the estimated mean difference of the magnitude of FC between the two stages and red dotted lines indicate the 95% confidence interval based on standard ANOVA analysis. The dark dot-dashed line in each plot denotes a sequence of coefficient estimates for quantile regressions with quantiles ranging from 0.05 to 0.95. The gray filled area surrounding the dot-dashed line indicates the 95% confident interval. The ANOVA results showed that the magnitude of FC did increase in the significant fatigue stage for all the six ROIs in the BG. All the tests using the standard ANOVA model were significant (*P* < 0.05 after Bonferroni correction). The quantile regression analysis revealed richer detail regarding how the FC changed in the individual quantiles and suggested a heteroscedastic nature of the FC alterations associated with muscle fatigue. Our results suggested that fatigue has more significant impact on upper quantiles of the magnitude of FC compared to the lower quantiles in left caudate, bilateral putamen, and left pallidum. For right caudate and right pallidum, fatigue has more significant impact on lower-to-medium quantiles of FC compared to the higher quantiles. The results illustrated that the areas with higher magnitude of FC in these ROIs were more synchronized with the seed area under the severe fatigue condition. These effects would be overlooked if only the standard ANOVA analysis was performed.

**Figure 3:**
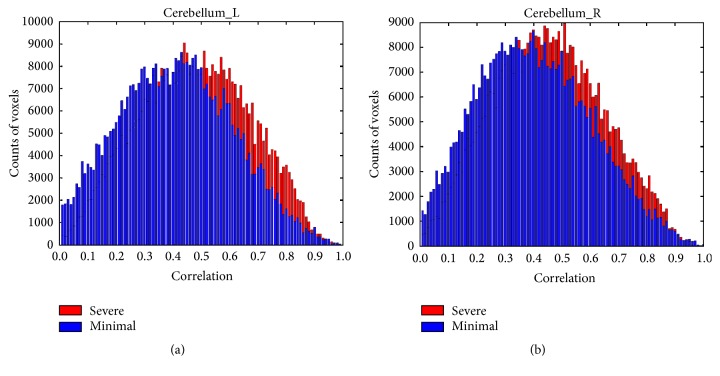
Comparisons of histogram plots of correlation coefficients over all subjects in cerebellum. Blue bins = data for* minimal* fatigue stage. Red bins = data for* significant* fatigue stage. Voxels with very low correlation (*r* < 0.001) were excluded for better visualization of coefficient distribution of the voxels in each ROI. In the significant fatigue stage, a larger number of voxels (red bins) can be seen with higher correlation (FC with seed area) values in mid-to-high correlation range in bilateral cerebellum ROIs.

**Figure 4:**
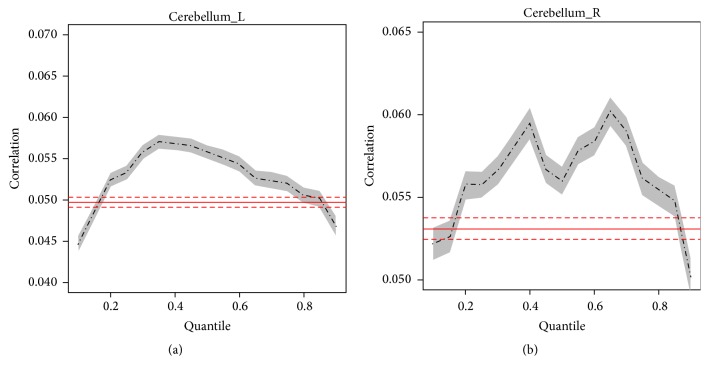
Results of quantile analysis from comparisons of coefficients of cross-correlation (FC) between minimal (first 100 s) and significant (last 100 s) fatigue stages in bilateral cerebellum. Comparisons were made at each quantile of the distribution curve between minimal and significant fatigue conditions in bilateral cerebellum. ANOVA results were plotted in red (solid line = mean; dotted line = 95% confidential intervals).

Histogram plots of FC data in CB also revealed a similar “shift” to higher correlation for both left and right CB in significant fatigue stage with significantly more voxels emerging on right tail (higher *r* values) of the histograms for both cerebella hemispheres in significant (last 100 s) compared to minimal (first 100 s) fatigue stages (Figure 3). Quantile regression analysis further verified that the FC increased significantly in significant compared with minimal fatigue stages for most of the quantiles from *r* = 0.2 to *r* = 0.8 in bilateral CB. The right CB showed greater FC with the M1 compared to the left CB as shown in Figure 4.

**Figure 5:**
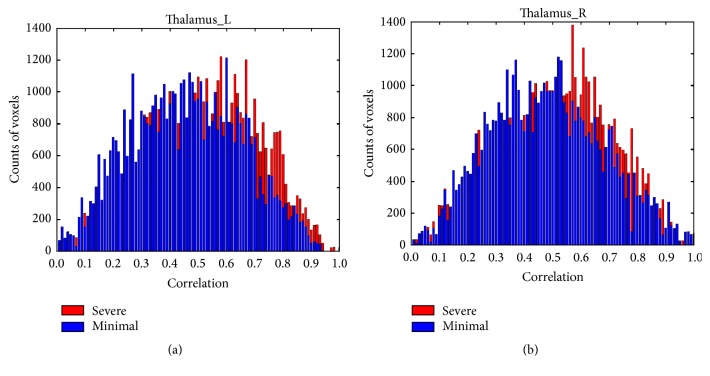
Comparisons of histogram plots of correlation coefficients over all subjects in bilateral thalamus. Blue bins = data for* minimal* fatigue stage. Red bins = data for* significant* fatigue stage. Voxels with very low correlation (*r* < 0.001) were excluded for better visualization of coefficient distribution of the voxels in each ROI. More voxels (red bins) can be found in the significant fatigue stage to have higher correlation (FC with seed area) values in low-to-high correlation range in bilateral thalamus ROIs.

**Figure 6:**
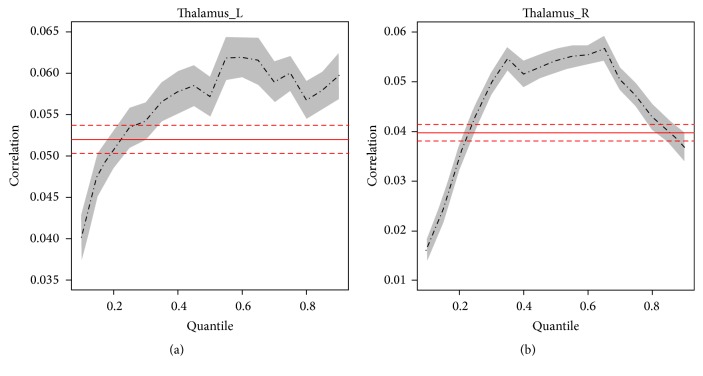
Results of quantile analysis from comparisons of coefficients of cross-correlation (FC) between minimal (first 100 s) and significant (last 100 s) fatigue stages in bilateral thalamus. Comparisons were made at each quantile of the distribution curve between minimal and significant fatigue conditions in bilateral thalamus. ANOVA results were plotted in red (solid line = mean; dotted line = 95% confidential intervals).

In both left and right TL, a similar “shift” to higher correlation in histogram was observed in significant fatigue stage with significantly more voxels emerging on right tail (higher *r* values) compared with the minimal fatigue stage (Figure 5). Quantile regression analysis confirmed that this “shift” of FC reached statistical significance in significant (*P* < 0.05) compared to minimal fatigue stages for most of the quantiles from *r* = 0.2 to near *r* = 1.0 in bilateral TL nuclei as shown in Figure 6.

## 4. Discussion

Muscle fatigue is associated with force loss due to fatigue of the active motor units. In MVC task, fatigue is accompanied by continuing force loss from the initial level. In a submaximal force task, the target force can be maintained for a period of time depending on the level of the target and the fatigue-induced force loss is compensated by a progressively increased effort to recruit additional motor units and/or increasing activation (discharge rate) level of not-yet-fatigued participating motor units to generate extra force. Although physiological factors during the fatigue process at muscular level have been studied extensively, there is very limited knowledge regarding how the motor control network at cortical and subcortical levels regulates the fatigue process and/or the network activities are affected by fatigue occurring in the muscle.

In the present study, we investigated the effect of muscle fatigue on functional connectivity (FC) between the contralateral M1 and subcortical motor control structures (BG, CB, and TL) during a submaximal handgrip motor task using quantile regression. We found significant increases in FC in the bilateral CB, BG, and TL towards the end of the task when significant fatigue occurred compared to the FC at the start of the task (minimal fatigue). These increased interregional correlations implicate perhaps underlying unified modulation among regions in the motor control network across cortical and subcortical sites presumably to counterbalance the diminishing force production capacity of the fatigued motor units. To the best of our knowledge, this is the first report on cortical-subcortical FC reflecting intensified synchrony among the cortical and subcortical regions in modulating high-demanding muscle output involving significant fatigue.

### 4.1. Functional Neuroimaging of Fatigue

Although fMRI has increasingly been used to characterize brain activation adaptation in populations with various neurological and neuropsychological disorders, its application in central modulation of fatigue is still in its infancy [17]. Only a few published studies have been discovered in our literature search. Studies have attempted to map cortical activities during process of voluntary muscle fatigue and reported progressive increase of fMRI signal-indicated brain activation in association, secondary, and primary motor cortices [1, 18]. Another study used Granger causality analysis (this is a different approach from our FC analysis; Granger causality infers directionality from the observed interregional correlations while FC simply measures the magnitude of the synchronization between regions) to explore the causal relationship based on fMRI data between various brain motor network nodes including M1 and CB. The authors of [19] found a disconnected hierarchical motor network due to the presence of muscle fatigue. This finding is interesting as it showed impaired connectivity between a higher-order and a lower-order cortical field during muscle fatigue. However, the graph model employed in their analysis was oversimplified (only 6 ROIs were used without differentiating laterality) and no subcortical regions except CB were included in their network model. Furthermore, one should be cautious in interpreting results from Granger causality analysis because of the limited time resolution of the fMRI data (usually around 2 s) comparing with the time scale of interregional brain modulation (events on millisecond level). A more recent fMRI study examined FC in the brain during muscle fatigue and reported strengthened FC among regions forming cortical motor control network [3]. So far the relationship between cortical and subcortical motor control centers in modulating muscle fatigue has not been well studied despite the important role shared between the two levels that form an integrated control system [6]. In the current study, we analyzed FC in the subcortical regions (BG, CB, and TL) with contralateral M1 (seed ROI) to examine dynamic activation relationship between them under the condition of voluntary muscle fatigue. Voluntary motor actions are controlled by a network consisting of not only cortical sensorimotor areas including primary sensory cortices (S1) and primary motor cortices (M1), secondary motor fields including premotor cortex (PMC) and supplementary motor area (SMA), and association cortices such as prefrontal, cingulate, and parietal regions but also subcortical centers including the CB, BG, and TL. Knowledge of roles of the subcortical regions during fatigue in relation to those of the cortical areas is of great importance for a better understanding of modulation of prolonged motor performance by the CNS network. The knowledge would help gain insights into mechanisms of fatigue syndromes in many clinical populations. For this reason, we chose to investigate FC between activation time course signals of the BG nuclei, CB, and TL with the contralateral M1 under minimal and significant fatigue conditions. The BG, TL, and CB play important roles in controlling voluntary movements [6] and they can be reliably segmented with high precision from high-resolution MR images [20].

### 4.2. Modulation of FC in Basal Ganglia

The BG is comprised of a group of nuclei found bilaterally in deep brain, which are known to be associated with a variety of functions such as voluntary movement and cognition [21]. Disruption in functions of the basal ganglia can lead to movement disorders such as Parkinson's and Huntington's diseases [22]. Using the quantile regression tool, we found significant fatigue-related FC increases in bilateral BG subregions including putamen, caudate, and pallidum in healthy young individuals. This finding is in line with mounting evidences of the BG's involvement in fatigue modulation. Since BG is also important for cognitive function [23], mediating cognitive-motor interaction in resisting muscle fatigue is a good example of cognitively regulating a demanding motor action.

Patients with Parkinson's disease (PD) are known to be prone to fatigue symptoms and this provides a good opportunity to study the possible link between fatigue and the BG [24]. Fatigue symptom in patients with other diseases may also have its root in the BG. For example, fatigue is a common complaint in patients with multiple sclerosis (MS). An imaging study [25] carried out in MS patients found significant hypometabolism (decreased metabolic activity) in the BG (from the rostral putamen to lateral head of the caudate nucleus). The observed fatigue symptom in MS thus likely is due to abnormal activation as a consequence of hypometabolism in the organ. Fatigue symptoms induced by radiation and/or chemodrugs during chemotherapy or radiotherapy in cancer patients were found to be related to the dosage of radiation to a variety of brain regions including the BG [26].

We found unified increase of FC in all six subregions of the BG examined: bilateral caudate, putamen, and pallidum. The caudate is linked to reward and can modulate the oculomotor outputs based on visual information received [27]. In MS patients, the caudate was also found to show increased fMRI activation related to a processing-speed task when compared to healthy controls after induction of cognitive fatigue [17]. The putamen, another major nucleus of the BG, was found in an earlier study [28] to have less benzodiazepine (a neurotransmitter) in patients with PD. This might help explain the abnormal motor performance presenting in PD. Shape analysis [29] of both putamen and caudate in patients with traumatic brain injury and healthy controls suggested possible atrophy in both putamen and caudate, which was linked to poorer performance in a task-switching task. The pallidum is comprised of dorsal pallidum (also known as globus pallidus) and ventral pallidum and there is evidence supporting that symptom of chronic fatigue syndrome is correlated to the abnormal activity of the right globus pallidus during a reward-processing task [30].

In sum, our findings support the idea that the BG is actively involved in regulating motor fatigue in a coherent fashion with the M1. This suggests that the BG plays an important role in helping sustain prolonged muscle activities and perhaps resisting fatigue within the cortical-subcortical motor control network. This role is likely accomplished through a cognitive process of inputs from multiple sources and by helping the M1 to adjust the motor command. It is of great interest to see if patients with clinical fatigue syndrome such as MS-, cancer-, and PD-related fatigue preserve a similar level of FC between the BG and M1 as demonstrated by healthy individuals in this study. If not, this might suggest that strong FC and a room for the FC to be elevated with increased demand for greater muscular recruitment and activity level are critical to resist fatigue in long-duration motor acts.

### 4.3. Modulation of FC in Cerebellum

The CB's most established role relates to motor control. It contributes to motor planning and fine tuning of motor output through integration of inputs from peripheral and spinal cord afferents and efferent from the cortex ([9], [16, (chapter 42)]). We demonstrated in an earlier fMRI study that activation level related to maintaining a submaximal handgrip force in bilateral cerebellum increased progressively with finger flexor muscle fatigue [1]. A recent fMRI study on patients with MS has revealed increased activation in the left CB when patients underwent a right hand finger tapping task that led to fatigue [31]. The observed increase in FC in bilateral CB with the M1 indicates more synchronized activities between the two structures. This observation may explain the reported depletion of glycogen in rat CB after running for 120 min [32]. During prolonged exercises, more energy supply is needed to support stronger activation in CB and other motor control fields and synchronized activation among these regions in the control network. Furthermore, enlarged and wilder force variability [33] as fatigue progresses needs more dynamic adjustment by the CB as part of the overall central effort in the control network to cope with fatigue and maintain performance accuracy.

### 4.4. Modulation of FC in Thalamus

The TL is considered as a critical relay station for information originating from a variety of sources including the brain, BG, CB, and peripheral receptors through the spinal cord afferent pathway. There has been evidence of involvement of the TL in regulating fatigue. For example, activity in the contralateral TL was found to be negatively correlated to the fatigue severity in MS patients in a human fMRI study [34]. A more recent fMRI study [35] examined the role of the TL while subjects performed intermittent isometric handgrip contractions. The authors found significantly increased activation in the contralateral TL towards the task failure (indicating significant fatigue) compared to a time with no apparent sign of fatigue. Our finding of increased FC between the contralateral (left) M1 and bilateral TL is in line with earlier literature. The ventral lateral TL nucleus, which projects to the M1, might play an integrative role together with M1 in modulating muscle fatigue. Through projection to the inferior parietal and premotor cortices, the ventral anterior TL nucleus might also play a role in motor control especially under a stressful condition such as pain [36] or tiredness as a consequence of significant muscle fatigue. Considering the role of the TL in processing information such as pain, it is reasonable to speculate that the increased FC between the TL and M1 reported in the current study relates to handling stressful situations taking place in the peripheral such as maintaining joint stability or preventing injury caused by prolonged exercise.

### 4.5. Limitations

Several caveats to our FC methods are worth noting in this study. First, we restricted our subcortical FC analysis to a few structures. Other subcortical regions might also be involved in fatigue modulation. However, the BG, CB, and TL are major subcortical structures in the “motor control loop” that is essential for normal voluntary movements. Second, we placed the seed in the contralateral M1. A disadvantage to this method was that we could only examine the pairwise connections with the left M1. Presumably, modulation of fatigue also involves interactions between regions not involving our seed region. For example, we did not examine FC within the basal ganglia structure which was found to be associated with functional deficit in patients with PD [37]. Nevertheless, it is expected that the FC would increase with fatigue among regions within the basal ganglia given that all three tested BG substructures showed strengthened FC with the M1. Third, when the fMRI data were collected years ago, part of the cerebellum (inferior portion) was not placed into the acquisition field of view. For this reason, we could not segment the CB into subregions or ROIs reliably. Signal dropouts in deep brain structures such as TL and BG also prevented us from performing finer ROI analysis within those structures in the present study. However, the observation of substantially increased FC between the M1 and the subcortical structures with muscle fatigue, even at the whole organ level, provides new insights into the view of importance of synchronized effort by the entire motor control network, including cortical [3] and subcortical sensorimotor fields, to cope with stressful stimuli from the periphery. Fourth, due to the difficulty of recording muscle electrical signals online with fMRI data collection, we did not have the EMG data to show an increase in the descending command (represented by augmented EMG) in maintaining the same level of force in the fatigued state although we have previously shown parallel increases in fMRI signals in primary, secondary, and association motor control cortices along with increases in EMG level of muscles experience fatigue during a submaximal force motor fatigue task [38]. Numerous studies in the literature also report significant EMG increases in muscles sustaining a submaximal force till fatigue and the consensus is that the increase in EMG in sustaining the same force represents augmented effort or descending command in driving the motor neuron pool and muscle to compensate for fatigue-related force loss and prolong the performance. Finally, since we did not include a nonfatiguing task in our paradigm as a control task (i.e., subjects generating a very low force for the same number of trials or keeping the same force but increasing rest time between trials without causing fatigue), we cannot completely rule out the effect of time on the observed FC changes. However, based on our previous data this possibility is extremely low. In a study utilizing a 5% MVC handgrip as a control task for a muscle fatigue protocol, we did not see any significant changes in brain activation or EMG signal of working muscles throughout the duration of the low-force task, yet the fatigue task changed brain activation pattern dramatically [38]. Similarly, intermittent MVC handgrip contractions with a short intertrial interval or rest caused significant muscle fatigue and a shift of brain activation center but no such fatigue and shift occurred for a task with the same number of contractions but a much longer rest between trials [39]. These data [38, 39] suggest the increases in FC observed in this study were a result of muscle fatigue.

## 5. Conclusions

Functional connectivity (FC) between subcortical structures (BG, CB, and TL) and contralateral primary motor cortex (M1) was assessed to examine interactive modulation among the cortical and subcortical control centers in mediating muscle fatigue. Significant augmentation of FC was observed under muscle fatigue condition in the bilateral BG, CB, and TL with the M1. The increased activation synchronization between the M1 and subcortical (BG, CB, and TL) motor structures likely represents coordinated efforts among them in regulating fatigue, perhaps by analyzing feedback information from the fatiguing muscle and adjusting the descending command to optimize motor unit recruitment and activation level for extended motor performance.
